# Lattice flow diverter for the treatment of small and medium-sized anterior circulation aneurysms

**DOI:** 10.3389/fneur.2026.1729763

**Published:** 2026-01-20

**Authors:** Yuanjin Ma, Jian Long, Hongyan Hou, Wei Li, Xuexian Zhang

**Affiliations:** 1 Department of Interventional Medicine, The Second Affiliated Hospital of Wenzhou Medical University, Wenzhou, Zhejiang, China; 2 Department of Neurointervention, Jingmen People’s Hospital, Jingchu University of Technology Affiliated Jingmen People’s Hospital, Jingmen, Hubei, China; 3 Department of Medical Imaging, People’s Hospital of Zhanyi District, Qujing, Yunnan, China; 4 Department of Interventional Medicine, The First Affiliated Hospital of Guangzhou Medical University, Guangzhou, Guangdong, China

**Keywords:** anterior circulation, flow diverter, lattice, small and medium-sized aneurysms, treatment

## Abstract

**Background:**

The advent of flow diverters (FDs) has revolutionized the treatment of intracranial aneurysms. The Lattice Flow Diverter (LFD) is a novel, domestically developed FD in China. To date, no clinical reports have described the use of the LFD in treating small and medium-sized intracranial aneurysms. In this study, we aimed to evaluate the safety and efficacy of the LFD in the treatment of small and medium-sized aneurysms in the anterior circulation.

**Methods:**

We retrospectively reviewed patients with small or medium-sized anterior circulation aneurysms who underwent LFD implantation at Jingmen People’s Hospital between September 2023 and May 2025. Demographic data, aneurysm morphology, and procedural details were collected from complete clinical and imaging records. Periprocedural neurological complications, angiographic outcomes, and clinical follow-up results were systematically analyzed.

**Results:**

A total of 56 patients were included. Among 56 patients, nine patients (16.1%) underwent adjunctive coil embolization. Two patients (3.6%) experienced periprocedural complications, both minor ischemic strokes. During a mean clinical follow-up of 7.18 months, complete occlusion (OKM grade D) was achieved in 71.4% of aneurysms, and adequate occlusion (OKM grades C + D) in 85.7%. All patients had a favorable clinical outcome (mRS score 0–2). Subgroup analysis showed no statistically significant differences in complete or successful occlusion rates between patients treated with LFD alone and those treated with LFD combined with coil embolization (*p* > 0.05).

**Conclusion:**

Our study preliminarily suggests that the use of the LFD for small and medium-sized anterior circulation aneurysms is associated with acceptable periprocedural safety and favorable short term angiographic and clinical outcomes. Further large-scale, multicenter, prospective studies are required to validate these findings.

## Introduction

Intracranial aneurysms (IAs) are common cerebrovascular disorders characterized by focal pathological dilatation of cerebral arteries, with a global prevalence estimated at 3 to 5% (1, 2). Since the United States Food and Drug Administration (FDA) approved the Pipeline Embolization Device (PED; Medtronic, Irvine, CA) in 2011 (3), flow diverters have undergone more than a decade of continuous refinement. Owing to their high rates of aneurysm occlusion and low recurrence rates, FDs have become an established treatment modality for large and giant IAs (4).

Notably, large and giant aneurysms constitute only a small proportion of all IAs, whereas unruptured small and medium-sized IAs (≤12 mm) account for approximately 80% of cases (5). Moreover, most ruptured aneurysms are less than 10 mm in diameter, and the annual rupture rate of small aneurysms ranges from 0.5 to 2% (6). Anatomically, small and medium-sized aneurysms most frequently arise along the internal carotid artery (7).

The Lattice Flow Diverter, developed by AccuMedical (Beijing, China), is an innovative, domestically designed flow-diverting device that incorporates two pioneering technological innovations. First, a mechanically controlled balloon-assisted delivery system enables adjustable radial expansion and enhanced wall apposition, thereby reducing vessel injury associated with conventional push–pull deployment techniques. Second, a proprietary Metal Interface Reassembly for Optimizing Restenosis (MIROR) dual-function surface engineering technology synergistically minimizes thrombogenicity while promoting endothelial healing (8).

Although small and medium-sized aneurysms represent the majority of clinical cases and carry a definite risk of rupture, studies evaluating the use of FDs in this subgroup remain limited. To date, no reports have described the use of the LFD for the treatment of small or medium-sized IAs. Therefore, this study systematically analyzed clinical data from patients with anterior circulation small and medium-sized aneurysms treated with the LFD, aiming to evaluate its safety and efficacy and to provide practical evidence for its clinical application.

## Materials and methods

### Patient selection

This retrospective study included patients with small or medium-sized anterior circulation aneurysms who underwent treatment with the LFD at Jingmen People’s Hospital between September 2023 and May 2025. The study was approved by the institutional ethics committee. Given its retrospective design and the anonymization of all patient data, the requirement for informed consent was waived. The study was conducted in accordance with the principles outlined in the Declaration of Helsinki.

Inclusion criteria were as follows: (1) age between 18 and 80 years; (2) diagnosis of untreated, unruptured IA confirmed by Computed Tomography Angiography (CTA), Magnetic Resonance Angiography (MRA), or Digital Subtraction Angiography (DSA); (3) treatment of the target aneurysm using a single LFD; and (4) aneurysm located in the anterior circulation with a maximum diameter ≤12 mm.

Exclusion criteria included: (1) Ruptured IA; (2) Prior endovascular or surgical treatment of the target aneurysm; (3) Treatment of multiple aneurysms using a single FD; and (4) Incomplete clinical or imaging follow-up data.

### Antiplatelet management

All patients received Dual Antiplatelet Therapy (DAPT) consisting of aspirin (100 mg/day) and clopidogrel (75 mg/day) for 7 days prior to the procedure. Thromboelastography (TEG) was performed 1 day before the intervention to assess platelet inhibition and guide adjustments. In cases of clopidogrel resistance, ticagrelor (90 mg twice daily) was substituted postoperatively in combination with aspirin. Dual Antiplatelet Therapy was maintained for at least 3 months after the procedure, followed by lifelong aspirin monotherapy.

### Endovascular procedure

All endovascular procedures were performed under general anesthesia. After successful femoral sheath placement, systemic heparinization was initiated with a bolus dose of 50–70 U/kg, followed by an additional 1,000 U hourly during the procedure. A tri-axial system was used to access the target aneurysm via the right femoral artery.

A 6F NeuroMax long sheath (Penumbra, United States) was positioned at the origin of the parent artery, followed by advancement of a 6F Sofia intermediate catheter (MicroVention, United States) to the proximal cavernous segment of the internal carotid artery. A Sine 27 microcatheter (AccuMedical, China) was navigated to the distal parent artery over a Synchro 14 microwire (Stryker, United States).

If adjunctive coiling was required, a shaped Echelon 10 microcatheter (ev3, United States) was introduced into the aneurysm sac. The LFD delivery system was then advanced to the distal tip of the Sine 27 microcatheter. Radiopaque markers on the mechanical balloon (10 mm spacing when sheathed) were used for precise intraluminal measurement and positioning. The microcatheter was retracted until the distal marker aligned with the intended landing zone, and the device was gradually deployed. During deployment, the mechanical balloon automatically expanded, ensuring instantaneous opening and optimal wall apposition.

After complete release, the microcatheter was retracted to cover one to two balloon segments proximally, and a push-pull maneuver was performed to fold the balloon and enhance apposition—effectively replacing conventional wire massage or balloon angioplasty. Post-deployment, standard anteroposterior and lateral angiograms were obtained to evaluate aneurysm filling and parent artery patency.

Technical success was defined as successful device deployment, complete neck coverage, satisfactory wall apposition, and preserved parent artery flow.

### Data collection and follow-up

Clinical data were extracted from the hospital’s electronic medical records, including: Demographic characteristics (age, sex, presenting symptoms such as headache or dizziness, and history of hypertension, diabetes, or smoking); Aneurysm and procedural details, including location, size, morphology, and whether adjunctive coiling was performed.

Immediate postoperative angiography was used to assess technical success. Patients were advised to undergo DSA follow-up at 6 and 12 months post-procedure to evaluate aneurysm occlusion and clinical outcomes.

Aneurysm filling was graded according to the O’Kelly-Marotta (OKM) classification: Grade A (complete filling, >95%), Grade B (partial filling, 5–95%), Grade C (neck remnant, <5%), Grade D (no filling, 0%) Complete occlusion was defined as OKM grade D, and adequate occlusion as grades C or D (2). Occlusion status was independently evaluated by two senior neurointerventionalists with >10 years of experience, with discrepancies resolved by consensus. At discharge and during 6 and 12 months follow-up, data were collected on mortality, in-stent stenosis (ISS), and neurological complications (ischemic stroke or subarachnoid hemorrhage). Clinical outcomes were assessed using the Modified Rankin Scale (mRS), dichotomized as good (0–2) or poor (3–6) (10). In-stent stenosis was classified as follows: none (0%), mild (≤50%), moderate (51–75%), severe (>75%), or complete occlusion (8).

### Statistical analysis

Statistical analyses were performed using SPSS version 25.0 (IBM Corp., Armonk, NY, United States). Data normality was assessed with the Kolmogorov-Smirnov test. Continuous variables were expressed as Mean ± Standard Deviation (SD), and categorical variables as percentage value. Group comparisons for categorical data were conducted using the Pearson *χ*^2^ test or Fisher’s exact test, as appropriate. A *p*-value <0.05 was considered statistically significant.

## Results

### Patient and aneurysm characteristics

**Figure 1:**
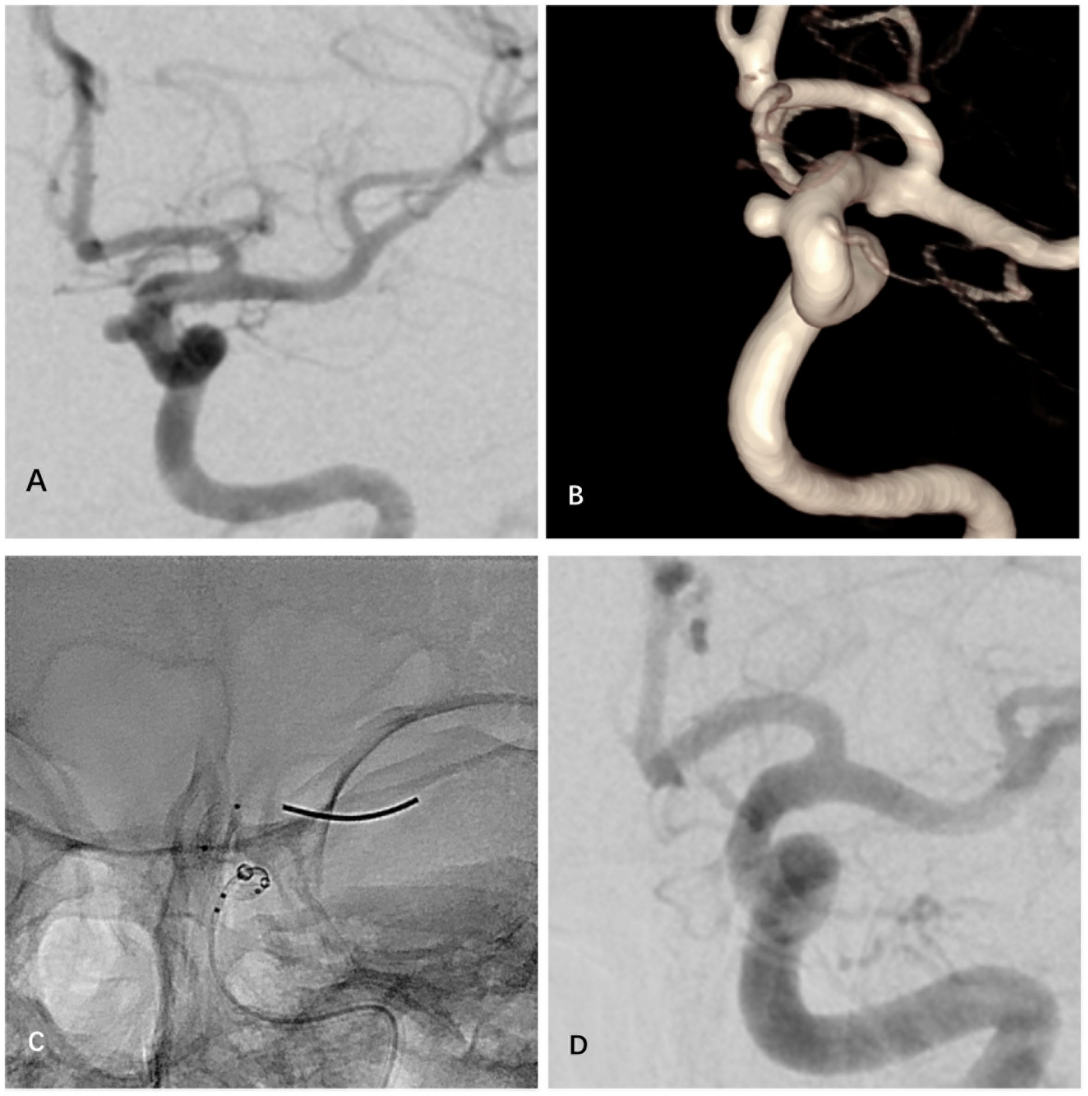
A case of unruptured small-to-medium aneurysm at the C6 segment of the left internal carotid artery treated with LFD. **(A)** Preoperative angiography showed a saccular aneurysm at the C6 segment. **(B)** Preoperative three-dimensional reconstruction revealed the accurate shape and size of the aneurysm. **(C)** Intraoperative deployment process of the LFD device. **(D)** The final angiographic follow-up showed complete occlusion of the aneurysm (OKM Grade D) without ISS.

Based on the selection criteria of this study, a total of 56 patients were finally included in the analysis, consisting of 16 males and 40 females with a mean age of 59.68 ± 8.63 years. All patients had a preoperative mRS score of 0. Admission etiologies included 24 cases with dizziness, 19 cases with headache, 1 case with limb numbness, and 12 cases with incidentally discovered IAs during physical examination. Twenty-four patients had a history of hypertension, 9 had diabetes mellitus, and 10 had a previous smoking history. All aneurysms were located in the internal carotid artery, with 2 in the C5 segment, 44 in the C6 segment, and 10 in the C7 segment. The mean maximum diameter of the aneurysms was 5.16 ± 2.20 mm, and the mean neck diameter was 3.96 ± 1.45 mm. All aneurysms were saccular in morphology. A total of 9 patients underwent adjuvant coil embolization. At discharge, all patients achieved an mRS score of 0–2. Detailed information on patient baseline data is provided in Table 1. Figure 1 depicts a case of a small to medium-sized anterior circulation aneurysm treated with LFD.

**Table 1:** Patients’ baseline data.

Variable	Frequency
Total patients (*n*)	56 (100%)
Demographic characteristics	
Gender	
Male	16 (28.6%)
Female	40 (71.4%)
Age (years)	59.68 ± 8.63
Symptom	
Dizziness	24 (42.9%)
Headache	19 (33.9%)
Limb numbness	1 (1.8%)
None	12 (21.4%)
Past medical history	
Hypertension	24 (42.9%)
Diabetes	9 (16.1%)
Smoking	10 (17.9%)
Endovascular procedure details	
Aneurysm location	
C5 segment of the ICA	2 (3.6%)
C6 segment of the ICA	44 (78.6%)
C7 segment of the ICA	10 (17.9%)
Aneurysm morphology	
Saccular	56 (100%)
Non-saccular	0 (0%)
Aneurysm measurements (mm)	
Aneurysm long diameter	5.16 ± 2.20
Aneurysm neck length	3.96 ± 1.45
Adjuvant coil embolization	
Yes	9 (16.1%)
No	47 (83.9%)
Pre-treatment mRS score	
0	56 (100%)
1–6	0 (0%)
mRS score at discharge	
0–2	56 (100%)
3–6	0 (0%)

ICA, Internal carotid artery; mRS, Modified Rankin Scale.

### Follow-up outcomes

The technical success rate was 100%, indicating that the overall deployment process was smooth and successful. Notably, neurointerventional physicians encountered no difficulties during deployment, and no additional adjunctive measures such as balloon dilation were required in any case. The mean follow-up duration was 7.18 months. At the final angiographic follow-up, the rate of complete aneurysm occlusion (OKM Grade D) was 71.4% (40/56), and the rate of successful occlusion (Grades C + D) reached 85.7% (48/56).

During the perioperative period, 2 patients (3.6%) developed minor cerebral infarction, with a mRS score of 1 at discharge; their mRS scores recovered to 0 at the final clinical follow-up. No perioperative subarachnoid hemorrhage occurred in any patient. Four patients (7.1%) developed mild ISS (≤50%); there were no cases of moderate/severe stenosis or parent artery occlusion, and all cases of ISS were asymptomatic (Table 2).

**Table 2:** Angiographic and clinical follow-up data.

Variable	Frequency
Follow-up time (months)	7.18
Aneurysm occlusion status at the last angiographic follow-up	
Complete occlusion [*n* (%)]	71.4% (40/56)
Successful occlusion [*n* (%)]	85.7% (48/56)
Complication	
Post-procedure cerebral infarction	2 (3.6%)
Post-procedure SAH	0 (0%)
Post-procedure ISS	4 (7.1%)
Symptomatic ISS	0 (0%)
mRS score at the last clinical follow-up	
0–2	56 (100%)
3–6	0 (0%)

SAH, subarachnoid hemorrhage; ISS, in-stent stenosis; mRS, Modified Rankin Scale.

Finally, a subgroup analysis was performed to compare aneurysm occlusion rates in patients receiving standalone LFD stents versus those receiving LFD stents in combination with coils. The results demonstrated that at the time of the last angiographic follow-up, although the rates of aneurysm complete occlusion and successful occlusion in the standalone LFD group were both lower than those in the LFD plus coil embolization group, no statistically significant differences were observed between the two groups (*p* > 0.05) (Table 3).

**Table 3:** Comparison of aneurysm occlusion rates between patients treated with LFD alone and those with LFD combined with coils.

Aneurysm occlusion status at the last angiographic follow-up	LFD alone (*n* = 47)	LFD plus coils (*n* = 9)	*p*-value
Complete occlusion [*n* (%)]	32 (68.1%)	8 (88.9%)	0.388
Successful occlusion [*n* (%)]	39 (83.0%)	9 (100.0%)	0.329

## Discussion

In this single-center retrospective study, the novel mechanically balloon-based LFD demonstrated favorable performance in the treatment of unruptured small and medium-sized anterior circulation aneurysms. The device showed remarkable efficacy, achieving complete and adequate occlusion rates of 71.4 and 85.7%, respectively, at the final angiographic and clinical follow-up. Meanwhile, the LFD exhibited an excellent safety profile, with a low incidence of neurological complications—only 3.6% of patients experienced minor perioperative cerebral infarction, and no cases of perioperative subarachnoid hemorrhage were observed. All patients achieved good clinical outcomes (mRS score 0–2). The incidence of ISS was also low, occurring in only 7.1% of patients. These patients had mild ISS and were asymptomatic. In addition, subgroup analysis revealed no statistically significant differences in the rates of complete or successful aneurysm occlusion between patients treated with LFD alone and those treated with LFD combined with coil embolization (*p* > 0.05).

The LFD device offers three key mechanical-balloon-based advantages. First, it is equipped with multiple radiopaque markers. The proximal marker can be aligned with the intended landing zone to facilitate accurate positioning, while the sequential markers allow real-time visualization of the deployment process, thereby reducing the risk of vascular injury. Second, the balloon assists in optimal stent expansion, ensuring proper apposition of the device to the vessel wall. Third, it provides enhanced stability to the delivery wire tip, minimizing the likelihood of sudden tip movement and potential vascular trauma. In addition, the LFD stent incorporates MIROR surface treatment technology, which helps reduce intra- and postoperative thrombus formation, promotes endothelialization, and lowers the long-term incidence of ISS (1).

Simple coil embolization and stent-assisted coil embolization have long been established as traditional and effective approaches for the treatment of small and medium-sized IAs. However, the process of advancing a microcatheter and deploying coils into the aneurysm carries a risk of rupture, particularly in small aneurysms (7). Flow diverters (FDs) obviate the need for catheterization within the aneurysm sac, thereby simplifying the procedure considerably (11, 12). In recent years, FDs have rapidly become a mainstay in the management of IAs because of their high occlusion rates and low incidence of complications, especially in large, giant, and morphologically complex aneurysms.

However, the application of FDs is not limited to large, giant, or complex aneurysms. Several investigators have evaluated their clinical value in the treatment of small and medium-sized IAs.

The PREMIER trial was the first prospective, multicenter study to assess the safety and efficacy of FD treatment in this population. In that study, 141 patients with 141 wide-necked small or medium-sized IAs were treated with the PED. Follow-up angiography and clinical assessments were completed in 97.9% (138/141) of patients at 12 months, revealing complete aneurysm occlusion (Raymond grade I) in 81.9% (113/138), and complete occlusion without significant parent artery stenosis (≤50%) in 76.8% (106/138). The combined rate of major complications and mortality was 2.1% (3/140) (13). Subsequently, the PLUS study, a large, multicenter, real-world investigation, further evaluated the safety and efficacy of PEDs for small and medium-sized IAs. This study included 652 patients harboring 754 aneurysms, of which 64.3% (485/754) underwent angiographic and clinical follow-up (mean duration, 8.26 ± 5.91 months). Complete occlusion (Raymond-Roy class I) was achieved in 82.5% (400/485) of aneurysms, and 81.4% (395/485) of these had no significant parent artery stenosis (≤50%). The overall rate of major complications and mortality was 3.2% (21/652) (6). With the development of newer generations of PEDs—Pipeline Flex and Pipeline Shield—their performance has continued to improve. Arai et al. (14) compared the safety and efficacy of three generations of PEDs in 102 patients (104 small and medium-sized internal carotid artery aneurysms). After a mean follow-up of 333.9 ± 255.0 days, complete occlusion (Raymond-Roy class I) was achieved in 92.3% (96/104) of aneurysms, with no cases of parent artery stenosis or major stroke.

Beyond the PED, Dibas et al. (15) reported the clinical outcomes of another flow-diversion device—the Surpass Evolve (Stryker, United States)—for the treatment of small and medium-sized IAs. In their study of 129 patients (135 aneurysms) with a median follow-up of 10.2 months, the complete occlusion rate was 77.1% (101/131), ISS ≥ 50% occurred in 8.8% (11/125), and retreatment was required in only 0.8% (1/125). Severe stroke occurred in 1.6% (2/129) of patients, and mortality was 0.8% (1/129). A meta-analysis (16) including 41 studies with 2,614 patients further supported these findings. Devices included the PED, Surpass (Stryker, United States), DERIVO (Acandis, Pforzheim, Germany), FRED (MicroVention, Aliso Viejo, CA, United States), Pipeline Shield (Medtronic, United States), and Silk (Balt Extrusion, France). The pooled complete occlusion rate at 12 months was 74.9% (95% CI, 69.6–79.8), and the overall rate of major adverse events was 7.8% (95% CI, 4.8–11.4). Another FD developed in China, the Tubridge (MicroPort, Shanghai, China), has also been evaluated for the treatment of small and medium-sized IAs. Xie et al. (7) included 57 patients with 77 aneurysms, classified into a small aneurysm group (39 patients, 54 aneurysms) and a medium aneurysm group (18 patients, 23 aneurysms). The mean follow-up durations were 6.8 ± 1.7 months and 8.6 ± 1.3 months, respectively. Minor ischemic stroke occurred in 6 patients (15.4%) in the small aneurysm group. Complete occlusion rates were 88.46% for small aneurysms and 81.82% for medium aneurysms. One patient (2.6%) developed asymptomatic mild ISS, and no intracranial hemorrhages were reported in either group.

The findings from various international and domestic studies on multiple FD devices—including the PED and Surpass Evolve from abroad and the Tubridge developed in China—consistently demonstrate that FDs exhibit stable safety and efficacy in the treatment of small and medium-sized IAs. Similarly, the latest domestically developed LFD evaluated in the present study showed comparable performance in the management of anterior circulation aneurysms, confirming its reliable safety and efficacy. These results further substantiate the therapeutic value of FDs in this subset of aneurysms.

Several previous studies have suggested that FD combined with coil embolization significantly improves the complete occlusion rate of large or giant IAs compared with FD alone (17–19). However, a recent meta-analysis (20) reported no statistically significant difference in favorable occlusion rates (OKM grades C-D) between FD combined with coils and FD alone for the treatment of large and giant aneurysms. In the context of small aneurysms, Wang et al. (21) found that the use of the PED with adjunctive coil embolization did not differ from PED alone in terms of aneurysm occlusion rate. In contrast, for medium, large, and giant saccular aneurysms, PED combined with coils significantly increased occlusion rates. Fuga et al. (22) compared PED with and without adjunctive coil embolization in small and medium-sized IAs and reported that, at 6 and 12 months follow-up, the complete occlusion rate was significantly higher in the combined group. Zhu et al. (8) reported on the safety and efficacy of the LFD for the treatment of IAs and conducted a subgroup analysis comparing LFD with adjunctive coil embolization versus LFD alone. The results showed no statistically significant difference in complete aneurysm occlusion rates between the two groups. Our study findings also demonstrated that there were no statistically significant differences in the rates of complete aneurysm occlusion and successful occlusion between patients treated with standalone LFD stents and those treated with LFD stents combined with coils. Taken together, these results suggest that, whether for large and giant aneurysms or small and medium-sized aneurysms, the benefit of combining FDs with coils in terms of aneurysm occlusion remains controversial.

Importantly, as a novel FD, the LFD appears to achieve comparable efficacy when used alone or in combination with coil embolization for IAs. Nonetheless, further studies directly comparing LFD with and without adjunctive coil embolization are warranted to confirm this conclusion.

Overall, these findings provide a basis for future studies and support the continued evaluation of LFD in clinical practice.

### Limitations

We acknowledge several limitations of this study. Although detailed parameters were collected regarding patient characteristics, aneurysm features, management strategies, angiographic and clinical follow-up, and occlusion assessment, the study design inherently limits the generalizability of our findings. First, this was a single-center, retrospective study with regional characteristics and without a control group, which precludes direct comparison with conventional treatment strategies or other FDs and may introduce geographic bias. Second, the relatively small sample size could have led to selection bias. Third, the follow-up duration was relatively short, preventing comprehensive evaluation of the long-term safety and durability of the device, particularly concerning aneurysm healing and delayed ISS. Finally, all aneurysms in this cohort were located in the anterior circulation, specifically within the internal carotid artery; therefore, the results may not be generalizable to small and medium-sized aneurysms in the anterior cerebral, middle cerebral, or posterior circulation territories.

## Conclusion

Despite the inherent limitations, this single-center retrospective study preliminarily suggests that the use of the LFD for small and medium-sized anterior circulation aneurysms is technically feasible and is associated with acceptable periprocedural safety, as well as favorable short-term angiographic results and clinical outcomes. Further large-scale, multicenter, prospective studies are required to validate these findings and to assess long-term outcomes.

## Data Availability

The raw data supporting the conclusions of this article will be made available by the authors, without undue reservation.
